# Family-homelessness and child absence from school: How data linkage research has identified and quantified the association in Wales, UK

**DOI:** 10.23889/ijpds.v9i5.2725

**Published:** 2024-09-18

**Authors:** Ian Thomas

**Affiliations:** 1 Administrative Data Research Wales; 2 Cardiff University

## Objective

Home and school experiences are linked. Though evidence exists quantifying the association between family-homelessness and school absences, it largely originates from the United States. Understanding the nature of this association in Wales, UK, is important given differing social, economic, and homelessness policy contexts when compared to the United States.

## Approach

Analysis included pupils attending state funded schools in a single local authority in Wales, during the academic years 2012/13 to 2015/16. Pupils were flagged as experiencing family-homelessness in an academic year if their family were supported by the housing service operating in the local authority area. Outcomes of interest were number of total, authorised, and unauthorised half-day-sessions absent per academic year. Pupils were observed over multiple academic years (N = 105,355 pupil-years), enabling both cross-sectional and longitudinal (panel) analyses.

## Results

On average, pupils in families accessing the housing service missed a total of 10 half-day-sessions more per academic year than pupils whose families were not accessing the service. Unauthorised half-day-sessions absent were double for pupils in families who had approached the housing service. After controlling for personal and area characteristics, rates of all absences were higher among pupils whose families accessed the service.

## Conclusions

This study represents the first published linkage of homelessness and education data in the UK. Given the apparent association between absenteeism and accessing housing support, changes to policy, practice, and law should be explored to facilitate co-operation between schools and housing services in Wales, and to protect children and their families from further disadvantage.

